# Carbapenemase-producing enterobacteriaceae recovered from a Spanish river ecosystem

**DOI:** 10.1371/journal.pone.0175246

**Published:** 2017-04-05

**Authors:** Núria Piedra-Carrasco, Anna Fàbrega, William Calero-Cáceres, Thais Cornejo-Sánchez, Maryury Brown-Jaque, Alba Mir-Cros, Maite Muniesa, Juan José González-López

**Affiliations:** 1 Department of Clinical Microbiology, Hospital Vall d'Hebron, Vall d'Hebron Institut de Recerca (VHIR), Universitat Autònoma de Barcelona, Barcelona, Spain; 2 Department of Genetics, Microbiology and Statistics, Faculty of Biology, University of Barcelona, Barcelona, Spain; Cornell University, UNITED STATES

## Abstract

The increasing resistance to carbapenems is an alarming threat in the fight against multiresistant bacteria. The dissemination properties of antimicrobial resistance genes are supported by their detection in a diverse population of bacteria, including strains isolated from the environment. The objective of this study was to investigate the presence of carbapenemase-producing Enterobacteriaceae (CPE) collected from a river ecosystem in the Barcelona metropolitan area (Spain). Identification of β-lactamases and other resistance determinants was determined as was the antimicrobial susceptibility profile. Moreover, screening of virulence factors, plasmid addiction systems, plasmid partition systems and replicon typing was performed. The results identified 8 isolates belonging to different species (*Escherichia coli*, *Enterobacter cloacae*, *Klebsiella pneumoniae*, *Klebsiella oxytoca*, *Raoultella ornithinolytica*). The most prevalent enzyme was KPC-2 (n = 6), followed by VIM-1 (n = 2) and IMI-2 (n = 1), whereas no OXA-48-type was detected. In addition, one strain was positive for both KPC-2 and VIM-1 enzymes. All the carbapenemase-encoding plasmids carried at least one plasmid addiction or partition system, being *vagCD* and *parAB* the most frequently detected, respectively. *E*. *coli* and *K*. *pneumoniae* isolates carried a low number of virulence-associated factors and none of the detected clones has previously been identified in the clinical setting. These findings support the high dissemination potential of the carbapanemase-encoding genes and reinforce the idea that the environment is another reservoir that may play an important role in the capture, selection and dissemination of carbapenem resistance genes.

## Introduction

The current therapeutic options to combat infections caused by the increasingly detected *Escherichia coli* and *Klebsiella pneumoniae* clinical isolates resistant to third and/or fourth generation cephalosporins are becoming increasingly fewer [1]. Carbapenems are usually the last resort to fight against these difficult-to-treat infections as, in most cases, these microorganisms also exhibit a multidrug resistance phenotype [2]. Unfortunately, Enterobacteriaceae strains resistant to carbapenems are emerging and compromising the outcome of patients receiving these drugs and posing a serious threat to the health care system [2].

Previous studies have revealed the presence of carbapenemase- and/or extended-spectrum β-lactamase-producing Enterobacteriaceae in samples collected from rivers, effluents and hospital sewage systems [3]. The enzymes detected include KPC, IMI, VIM, IMP, OXA-48-type and NDM, highlighting their carriage in *K*. *pneumoniae* and *E*. *coli* strains [3]. The presence of these resistant determinants in aquatic environments can be understood according to different scenarios: i) these niches constitute a reservoir of novel carbapenem resistance genes (e.g. KPC-2 homologs have been identified in the genera *Chromobacterium*, commonly isolated from aquatic environments) [4]; ii) resistant bacteria of human origin are released into the environment hence spreading their resistance genes into environmental species [5]; iii) carbapenemase-producing Enterobacteriaceae (CPE) detected not only in farms but also in companion pets and wild animals, can also reach the environment via manure [3].

Recently there has been great interest in tracing plasmids conferring drug resistance, particularly carbapenem resistance, as a result of the promiscuity of these genes. Their incredible mobilization ability has forced the introduction of accurate surveillance and control measures to hinder their spread, with special effort in the clinical setting. Over the past years plasmid characterization has mainly been based on identification of the plasmid incompatibility group [6]. More recently, however, the study of plasmid addiction systems (PAS), and to a lesser extent, plasmid partition systems (PPS), has gained relevance. These mechanisms ensure stable transmission of plasmids by post-segregational killing of plasmid-free daughter cells and driving plasmid positioning during bacterial division processes. Their role in plasmid maintenance may represent a potential key factor involved in their successful dissemination [7,8].

At present, there are few epidemiologic studies regarding CPE recovered from rivers. Some studies have been conducted in Portugal [9], US [10], Tunisia [11], India [12] and China [13,14], but none in Spain. Moreover, little attention has been directed towards the prevalence of PAS and PPS in carbapenemase-encoding plasmids. The aim of the present study was to explore the presence of CPE in the Llobregat river ecosystem, an important river in the Barcelona metropolitan area which is the most populous metropolitan area in the Mediterranean coast and hence with a high anthropogenic impact and scarce contribution of animal fecal contaminants [15]. Isolates and plasmids harboring these resistance elements were characterized in order to gain new insight into the factors supporting their dissemination worldwide.

## Materials and methods

### Collection of river samples and selection of carbapenem-resistant Enterobacteriaceae

Eleven sediment and 12 water samples were collected from the Sant Joan Despí station (UTM: 31T 420339 4578455) of the Llobregat river, Catalunya, Spain from May to December, 2014. This river receives anthropogenic impact via secondary effluents of municipal wastewater treatment plants and industrial effluents along the river course in the Barcelona metropolitan area [16] whereas the impact of animal fecal contaminants is very low and irregular [15]. River water was used without further processing while five g of sediment were homogenized in PBS (1:10). After settling, the resulting supernatant was used for bacterial isolation. Serial dilutions of water and of the sediment supernatant in PBS were inoculated on chromID® CARBA SMART chromogenic culture media (BioMérieux) for screening CPE. Plates were incubated at 37°C for 48 h in aerobic conditions. The colonies obtained were identified by MALDI-TOF (VITEK MS, BioMérieux) and those identified as Enterobacteriaceae were selected for further analysis. Differentiation between *Raoultella* species (*R*. *planticola* and *R*. *ornithinolytica*) was carried out by PCR as described previously [17].

### Antimicrobial susceptibility testing

Antimicrobial susceptibility to β-lactams of all the enterobacterial isolates recovered was studied by the disk diffusion method following EUCAST guidelines [18]. In addition, the MICs of meropenem, imipenem and ertapenem were determined by the Etest (BioMérieux) according to the manufacturer’s recommendations. Evidence suggestive of carbapenemase production was determined using EUCAST screening cut-off values (disk inhibition zones of <25 mm for meropenem and/or ertapenem, and/or <23 mm for imipenem; or MICs > 0.12 mg/L for meropenem and/or ertapenem and/or > 1 mg/L for imipenem).

Finally, the MIC of additional antimicrobials was also determined by the Etest in all the isolates. The compounds studied were: amoxicillin, amoxicillin/clavulanic acid, piperacillin/tazobactam, ceftazidime, cefotaxime, cefepime, cefoxitin, aztreonam, gentamicin, amikacin, nalidixic acid, ciprofloxacin, trimethoprim-sulfamethoxazole, fosfomycin and colistin. Interpretation of the results was performed according to the EUCAST clinical breakpoints when available [19]. Otherwise, CLSI breakpoints were used (e.g. cefoxitin and nalidixic acid) [20].

### Detection of antimicrobial resistance genes, virulence genes and the phylogenetic group

All the isolates selected were screened by means of PCR and sequencing analysis to detect the presence of the following carbapenemase genes: *bla*_OXA-48_, *bla*_VIM_, *bla*_KPC_, *bla*_NDM_, *bla*_IMI_, *bla*_IMP_. In addition, carriage of further resistance genes such as those encoding ESBLs (*bla*_TEM_, *bla*_SHV_, *bla*_CTX-M_), plasmid-mediated AmpCs (*bla*_DHA_, *bla*_AAC_, *bla*_ACT_, *bla*_FOX_, *bla*_CMY_), the *bla*_OXA-1_ gene, and plasmid-mediated quinolone resistance genes (*aac(6’)-Ib-cr*, *qnrA*, *qnrB*, *qnrS*) was also studied by PCR and sequencing. Individual amplifications (S1 Table) were performed with the exception of the plasmid-mediated *ampC* β-lactamase genes, which were assessed according to the previously described multiplex PCR [21]. Identification of the phylogenetic group (A, B1, B2, C, D, E or F) of the *E*. *coli* strains was similarly detected by PCR according to the most recent classification [22].

All *E*. *coli* strains were screened for the presence of 49 virulence factors (including 19 adhesins, 4 siderophores, 11 toxins, 6 capsule synthesis-associated genes, and 9 miscellaneous genes). Screening was performed by multiplex PCRs according to previously reported methodology [23]. In the case of *K*. *pneumoniae*, the presence of 9 virulence-associated genes was assessed by PCR. The set of factors included: the adhesin-encoding genes *fimH* (Type 1 fimbriae) and *mrkD* (Type 3 fimbriae), the capsule- and LPS-associated genes *wabG* and *uge*, the two genes *magA* and *rmpA* related to the mucoid phenotype, as well as other virulence-associated genes such as *ureA* (intestinal colonization), *allS* (liver infection) and *kfuBC* (iron uptake system) [24].

### Clonal relatedness

Clonality of the *E*. *coli* isolates was analyzed by pulsed-field gel electrophoresis **(**PFGE) as described previously (http://www.cdc.gov/pulsenet/pdf/ecoli-shigella-salmonella-pfge-protocol-508c.pdf). Data were analyzed using GelCompar II v.4.6 (Applied Maths, SintMartens-Latem, Belgium). Requisites for strains to belong to the same pulsotype were to have a similarity criterion above 85%, with a position tolerance of 1% and an optimization of 1%.

Multilocus sequence typing (MLST) identification was carried out for *E*. *coli*, *K*. *pneumoniae* and *E*. *cloacae* by means of PCR and sequencing according to the protocols specified at the web sites http://mlst.ucc.ie/mlst/dbs/Ecoli, http://bigsdb.web.pasteur.fr/klebsiella/klebsiella.html and http://pubmlst.org/ecloacae, respectively.

### Plasmid characterization

Identification of plasmid replicons was accomplished using the PCR-based replicon typing method as described previously [6,25,26]. The presence of PAS, according to previously reported methodology, was sought by PCR of 3 type I systems: Hok-Sok, PndA-PndC, SnrB-SnrC; and 5 type II systems: PemK-PemI, CcdA-CcdB, RelB-RelE, ParD-ParE, VagC-VagD [7]. The following genes encoding PPS were also determined by PCR: *parAB*, *parMRC* and *sopAB*. The primer sequences and annealing temperatures are shown in S1 Table.

Plasmid size was determined by PFGE of total DNA digested with S1 nuclease. Hybridization was then carried out to confirm all positive cases detected in the PCR screening. Specific DNA probes were obtained by PCR amplification with the same primers reported in S1 Table. The digoxigenin PCR DIG Probe Synthesis kit (Roche Diagnostics) was used to obtain digoxigenin-labeled probes. Hybridization and detection were performed according to the manufacturer’s instructions.

Transferability of the carbapenemase-containing plasmids (*bla*_VIM-1_, *bla*_KPC-2_, *bla*_IMI-2_) was attempted by conjugation at 37°C and 30°C using a kanamycin-resistant derivative of *E*. *coli* HB101 as recipient. Transconjugants were selected on Luria-Bertani (LB) agar plates supplemented with 300 μg/mL kanamycin and 200 μg/mL ampicillin. Transformations were then carried out for strains for which no transconjugant could be obtained. Plasmids were extracted by using the hot alkaline method [27] and were transformed by electroporation into electrocompetent *E*. *coli* HB101. Selection of transformants was done on LB agar plates containing 300 μg/mL kanamycin and 200 μg/mL ampicillin. Successful conjugation and transformation was confirmed by specific PCR amplification.

## Results

### Identification and characterization of carbapenem-resistant Enterobacteriaceae isolates

High density colonies grew on the CARBA SMART agar plates inoculated with the river and sediment samples. A total of 224 colonies were obtained: 50 from river water and 174 from sediment samples. However, only 8 (3.6%) isolates, all recovered from sediment samples, were found to be Enterobacteriaceae. The remaining isolates were: *Pseudomonas putida* (56%), *Aeromonas* spp. (26.5%), *Pseudomonas fluorescens* (7.5%), *Pseudomonas chlororaphis* (4%), *Pseudomonas aeruginosa* (3.5%), and other non-Enterobacteriaceae species (2.5%).

The molecular studies revealed that 5 isolates carried KPC-2 (3 *E*. *coli*, 1 *K*. *pneumoniae*, 1 *Enterobacter cloacae*), 1 strain was positive for VIM-1 (*R*. *ornithinolytica*) and 1 for IMI-2 (*E*. *cloacae*), whereas the remaining isolate (*Klebsiella oxytoca*) carried two carbapenemases: KPC-2 and VIM-1 (Table 1).

**Table 1:** Features of the CPE strains and their carbapenemase-carrying plasmids.

				PCR detection of antimicrobial resistance genes						Characterization of the carbapenemase-carrying plasmids							
Isolate	Species	Sampling date	MLST	Carbapenemases			Oxacillinase	Other resistance genes		Plasmid size	Inc groups			PAS			PPS
1-CAR	*E*. *coli*	2014/07/08	ST1434	*bla*_KPC-2_	—	—	*bla*_OXA-1_	*aac(6')-Ib-cr*	*qnrB6*	70 kb	IncN	—	—	*vagCD*	—	—	—
2-CAR	*E*. *coli*	2014/07/08	ST5001	*bla*_KPC-2_	—	—	—	—	—	48 kb	—	IncR	—	—	—	—	*parAB*
3-CAR	*E*. *cloacae*	2014/05/27	ST822	—	*bla*_IMI-2_	—	—	—	—	170 kb	—	—	IncFIB	—	—	—	—
4-CAR	*E*. *coli*	2014/07/08	ST216	*bla*_KPC-2_	—	—	—	*aac(6')-Ib*	—	48 kb	—	IncR	—	—	*parDE*	*ccdAB*	—
5-CAR	*R*. *ornithinolytica*	2014/05/27		—	—	*bla*_VIM-1_	*bla*_OXA-1_	*aac(6')-Ib*	*qnrB5*	70 kb	—	IncR	—	*vagCD*	—	—	—
6-CAR	*K*. *pneumoniae*	2014/05/27	ST634	*bla*_KPC-2_	—	—	—	—	—	97 kb	—	—	IncFIIK	*vagCD*	—	—	—
7-CAR	*E*. *cloacae*	2014/07/08	ST823	*bla*_KPC-2_	—	—	*bla*_OXA-1_	*aac(6')-Ib-cr*	*qnrB6*	70 kb	IncN	—	—	*vagCD*	—	—	—
8-CAR	*K*. *oxytoca*	2014/05/27		*bla*_KPC-2_	—	*bla*_VIM-1_	*bla*_OXA-1_	*aac(6')-Ib*^a^	—	60 kb	IncN	—	—	—	—	—	*parAB*

^a^, This strain was positive for the presence of the *aac(6')-Ib* gene although it was detected in a different plasmid of circa 240 kb.

No strain was positive for the presence of additional β-lactam resistance determinants (i.e. ESBLs and plasmidic *ampC*s). Contrarily, up to 4 strains belonging to 4 different species were positive for the *bla*_OXA-1_ gene. Five strains were positive for the aminoglycoside acetyltransferase gene (2 carried the *cr* variant) and 3 for the presence of the *qnrB* gene (two *qnrB6* and one *qnrB5* variants) (Table 1). IMI-2-producing *E*. *cloacae* (3-CAR) was the only strain which was negative for the presence of any of the additional resistance genes evaluated.

All 3 *E*. *coli* strains belonged to different pulsotypes (data not shown) and different STs: ST1434 (1-CAR), ST216 (2-CAR) and the newly described ST5001 (4-CAR) (Table 1). Regarding the phylogenetic group, all 3 strains belonged to group A. The ST identified for the *K*. *pneumoniae* strain was ST634, whereas two new STs, ST822 and ST823, were assigned to the *E*. *cloacae* strains (3-CAR and 7-CAR, respectively).

### Antimicrobial susceptibility profile of the strains

Susceptibility to the 18 antibiotics evaluated (11 β-lactams and 7 non-β-lactams) is shown in Table 2. All the isolates showed the expected hydrolytic profile according to the carbapenemase encoded whereas variable results were observed for the remaining antimicrobials (aminoglycosides, quinolones, trimethoprim-sulfamethoxazole and fosfomycin). Overall, all strains (100%) were resistant to amoxicillin, amoxicillin/clavulanic acid and ertapenem. Most strains (n = 7, 88%) were non-susceptible to piperacillin/tazobactam, ceftazidime, cefotaxime, cefepime, aztreonam and imipenem; followed by 5 strains (63%) non-susceptible to meropenem. Thus, ertapenem was the least active carbapenem (MIC_50_ of 24 μg/mL and MIC_90_ of 32 μg/ml) whereas meropenem and imipenem were slightly more active (MIC_50_ of 4 μg/mL and MIC_90_ of 32 μg/ml, and MIC_50_ of 4 μg/mL and MIC_90_ of 12 μg/ml, respectively).

**Table 2:** Minimum inhibitory concentrations (MIC) of the studied strains.

	MICs (μg/mL)^b^											
Antimicrobials^a^	Environmental strains								Recipient strain	Transconjugants/Transformant		
	1-CAR	2-CAR	3-CAR	4-CAR	5-CAR	6-CAR	7-CAR	8-CAR		TC-1-CAR^e^	TC-7-CAR^f^	TF-4-CAR^g^
	*E*. *coli*	*E*. *coli*	*E*. *cloacae*	*E*. *coli*	*R*. *ornithinolytica*	*K*. *pneumoniae*	*E*. *cloacae*	*K*. *oxytoca*	*E*. *coli-*HB101^d^	*E*. *coli-*HB101	*E*. *coli-*HB101	*E*. *coli-*HB101
	KPC-2	KPC-2	IMI-2	KPC-2	VIM-1	KPC-2	KPC-2	KPC-2, VIM-1		KPC-2	KPC-2	KPC-2
AMP	**>256**	**>256**	**>256**	**>256**	**>256**	**>256**	**>256**	**>256**	2	**>256**	**>256**	**>256**
AMC	**48**	**48**	**128**	**>256**	**48**	**128**	**96**	**96**	2	**32**	**32**	24
TZP	**>256**	**32**	4	**>256**	**>256**	**192**	**>256**	**>256**	1.5	**>256**	**>256**	**>256**
FOX^c^	**64**	**192**	**>256**	**>256**	**>256**	24	**>256**	**48**	3	24	24	**32**
CAZ	4	**6**	0.25	**6**	**>256**	**8**	4	**>256**	0.064	4	**6**	**2**
CTX	**6**	**6**	0.25	**8**	**32**	**6**	**16**	**48**	0.064	**8**	**6**	**3**
FEP	**8**	**6**	0.94	**16**	**24**	3	**6**	4	0.047	**5**	4	2
ATM	**32**	**32**	**>256**	**>256**	0.5	**192**	**16**	**48**	0.016	**48**	**48**	**12**
IPM	3	1.5	**≥32**	**12**	**8**	4	4	3	0.19	4	4	2
MEM	4	1.5	**≥32**	**≥32**	1.5	**12**	1.5	4	0.016	2	1.5	0.5
ERT	**≥32**	**24**	**≥32**	**≥32**	**2**	**≥32**	**1.5**	**4**	0.002	**1.5**	**1.5**	**2**
GEN	0.38	0.38	0.25	1.5	1.5	0.19	1.5	0.38	0.19	0.5	1	0.25
AMK	3	1.5	2	4	1.5	1	12	2	0.75	4	12	0.75
NAL^c^	**256**	**>256**	4	12	6	16	**>256**	**>256**	2	4	4	4
CIP	**2**	0.38	0.125	0.75	0.5	0.75	**16**	**2**	0.002	0.064	0.064	0.006
SXT	0.19	0.19	0.94	0.19	0.19	0.19	0.38	**>32**	0.094	0.032	0.032	0.064
FOF	1	8	**>1024**	**384**	**>1024**	**1024**	16	**>1024**	2	4	2	1.5
CST	0.047	0.032	0.047	0.094	0.094	0.064	0.032	0.064	0.094	0.094	0.094	0.125

^a^, AMP, ampicillin; AMC, amoxicillin/clavulanic acid; TZP, piperacillin/tazobactam; CAZ, ceftazidime; CTX, cefotaxime, FEP, cefepime; ATM, aztreonam; IPM, imipenem; MEM, meropenem; ERT, ertapenem; GEN, gentamicin; AMK, amikacin; CIP, ciprofloxacin; SXT, trimethoprim-sulfamethoxazole; FOF, fosfomycin; CST, colistin.

^b^, Numbers in bold-face indicate resistance values according to EUCAST.

^c^, Bold-face numbers for cefoxitin and nalidixic acid refer to resistance values according to CLSI breakpoints as no value is reported in EUCAST guidelines.

^d^, *E*. *coli* HB101 was the strain used as recipient in the conjugation and transformation experiments.

^e^, *E*. *coli* transconjugant obtained from strain 1-CAR and HB101 that received *bla*_KPC-2_-encoding plasmid.

^f^, *E*. *coli* transconjugant obtained from strain 7-CAR and HB101 that received *bla*_KPC-2_-encoding plasmid.

^g^, *E*. *coli* transformant obtained from strain 4-CAR and HB101 that received *bla*_KPC-2_-encoding plasmid.

Regarding non-β-lactam compounds, all strains (100%) were susceptible to gentamicin and colistin, 7 (88%) to amikacin and trimethoprim-sulfamethoxazole, whereas 5 strains (63%) were non-susceptible to ciprofloxacin and fosfomycin.

### Detection of virulence genes

With respect to the analysis of the virulence-related genes, the three *E*. *coli* strains collected to study for presence of 49 virulence-related factors were only positive for the type I fimbrial adhesin *fimH*.

In the case of the *K*. *pneumoniae* strain, it was positive for the two fimbrial adhesin-encoding genes *fimH* (type I fimbriae) and *mrkD* (type 3 fimbriae), the two genes related to LPS and capsule biosynthesis *wabG* and *uge*, as well as *ureA*, the ureasa A subunit related to colonization.

### Characteristics of the carbapenemase-encoding plasmids

Replicon typing analysis (Table 1) revealed that there was variability for the type of carbapenemase-encoding plasmids encountered in terms of incompatibility group and size. Specifically, for the 5 strains only positive for KPC-2, this resistance determinant was located in an IncN (70 kb), IncR (48 kb) or IncFIIK (97 kb) plasmids. In the case of the two VIM-1-producing microorganisms, this gene was carried in an IncR (70 kb) or IncN (60 kb) plasmids, being the latter also positive for KPC-2. Lastly, the results showed that the IMI-2-positive strain carried this resistance determinant in a larger, IncFIB plasmid (170 kb).

The analysis of PAS of the carbapenemase-encoding plasmids detected in the present study revealed that VagCD was the most prevalent system, being present in 4 (50%) strains (*E*. *coli* 1-CAR, *R*. *ornithinolytica* 5-CAR, *K*. *pneumoniae* 6-CAR, *E*. *cloacae* 7-CAR), followed by the ParDE (12.5%) and CcdAB (12.5%) systems which were codetected in the same strain (*E*. *coli* 4-CAR) (Table 1). On the contrary, three strains (*E*. *coli* 2-CAR, *E*. *cloacae* 3-CAR, *K*. *oxytoca* 8-CAR) were negative for the presence of all of the systems studied. The results obtained for the presence of PPS revealed that only two (25%) strains (*E*. *coli* 2-CAR, *K*. *oxytoca* 8-CAR), which were negative for PAS, carried the *parAB* genes. Only the IMI-2-producing *E*. *cloacae* (3-CAR) strain was negative for all the PAS and PPS evaluated. These results indicate that none of the plasmid-related stability systems studied here was associated with a particular carbapenem-resistance mechanism.

Regarding plasmid mobilization experiments, *bla*_KPC-2_-encoding transconjugants could be obtained from strains 1-CAR and 7-CAR. Additionally, a *bla*_KPC-2_-encoding tranformant was recovered from strain 4-CAR. The MICs for different antibiotics were evaluated for receptor and transconjugant/transformant strains and are shown in Table 2. Of note, the MICs of ERT and FOX were much lower in recipient strains than in donor strains. These changes might be due to the presence of chromosomally-encoded resistance mechanisms, as outer membrane permeability alterations, in the donor strains affecting the susceptibility to both antibiotics.

## Discussion

The increasing rates of resistance to antimicrobial compounds that play a key role in the clinical setting, such as carbapenems, have led to an urgent need to understand the global spread of their resistance mechanisms. As a result, the role and impact of other ecological niches, such as rivers and hospital sewage systems, is of important concern, as is the detection of β-lactamases and other unrelated resistance mechanisms in these strains.

The CPE identified in the present work were only recovered from sediment samples. These results are in agreement with recent works which report increased load and longer persistence of bacteria inhabiting sediment samples in comparison with river water samples. Thereby these results suggest higher probability for detecting CPEs in sediment than river water [28]. Accordingly, the CPE isolated in this study were *E*. *coli* (3 strains, positive for KPC-2), *K*. *pneumoniae* (KPC-2-producer), *K*. *oxytoca* (KPC-2- and VIM-1-producer), *E*. *cloacae* (one positive for KPC-2 and one positive for IMI-2) and, lastly, *R*. *ornithinolytica* (VIM-1-producer). In terms of environmental epidemiology, the most prevalent microorganism producing KPC-2 is *Klebsiella*, followed by *Enterobacter* [5], albeit other microorganisms such as *E*. *coli* and *Raoultella* spp. have also been reported [5,29]. The VIM-1 enzyme has occasionally been detected in environmental samples and in a narrow spectrum of genera (e.g. *K*. *pneumoniae* [11] and *E*. *coli* [9]). There is only one previous identification in a *R*. *ornithinolytica* strain collected from fecal carriers in Spain [30] whereas, to our knowledge, this is the first identification of *bla*_VIM-1_ in this bacteria isolated from the environment. On the contrary, only three IMI-2 identifications have been documented so far: one *Enterobacter asburiae* (river sample, US) [10], one *E*. *cloacae* (clinical isolate, China) [14], and one *E*. *coli* (clinical isolate, Spain) [31]. Thus, this is also the first time that the IMI-2 determinant has been detected in bacteria of environmental origin in Europe.

Despite the increasing rates of carbapenem resistance in the clinical setting, it is rather infrequent to codetect several carbapenemases in the same strain. The most frequently identified microorganism is *K*. *pneumoniae* with a percentage estimated at 5.3% on considering carbapenemase-positive isolates in Greece, a carbapenemase endemic country [32]. In the present study we have codetected *bla*_VIM-1_ and *bla*_KPC-2_ in the same IncN plasmid in a *K*. *oxytoca* strain, being this the first case reported from an environmental strain.

In a multicenter study conducted in 2013 it was shown that the most prevalent carbapenemase in Enterobacteriaceae in Spain was OXA-48 (n = 271; 71%), followed by the VIM-type enzymes (n = 96; 25%), whereas the KPC family was uncommon (2%), and no isolate was an IMI-type producer [33]. Surprisingly, in the present study, in which the isolates were recovered only one year later, no OXA-48 was detected and VIM-type carbapenemases were represented at a lower frequency than KPC-type enzymes. Previous results available in the literature also describe a major prevalence of KPC and VIM determinants in the aquatic environment [3,9,12,29]; whereas, to our knowledge, no report has detected an OXA-48-producing CPE in the same type of samples.

The characterization of the virulence potential of the *E*. *coli* strains detected in this study revealed that they only were positive for *fimH* and belonged to the phylogenetic group A (usually associated with commensal strains that carry very few virulence factors). Three STs were identified in *E*. *coli*: i) ST1434, previously detected in Mexico in two strains recovered from water and human feces which were categorized as non-pathogenic [34]; ii) ST216, identified among ESBL-producing *E*. *coli* strains of fecal origin in the US [35] and Europe [36], and as the most prevalent ST in IMP-producing strains recovered from gulls feces in Australia [37]; and iii) ST5001, a singleton newly described in this study. Both ST1434 and ST216 belong to clonal complex CC10, from which several KPC-producing isolates have previously been described, although only in surveillance culture studies in Israel [38]. Concerning *K*. *pneumoniae*, even though strain 6-CAR possessed the most prevalent virulence factors reported in clinical isolates (*fimH*, *mrkD*, *wabG*, *uge* and *ureA*) [24], it was negative for the genes most significantly associated with invasive human infection (*rmpA*, *fyuA*, *iutA*, *clbA-R*, *iroN*) [39]. This strain belonged to ST634, a singleton for which no previous information could be found. Altogether, this information suggests that the virulence potential of the *E*. *coli* and *K*. *pneumoniae* isolates found in the present study may be considered of low risk. In the case of the two *E*. *cloacae* isolates, both belonged to STs of new description.

Plasmid addiction and partition systems have not been studied in depth when characterizing clinically-relevant plasmids. *vagCD* has been reported as the most represented PAS in clinical samples related to the spread of β-lactamases, followed by *ccdAB*, *pemIK* and *hok-sok* [40]. Concerning PPS, *parAB* has also been associated with carbapanemase-producing plasmids, as in the case of the widespread pOXA-48a plasmid in *K*. *pneumoniae* [41]. Similar results were detected in the present study: *vagCD* and *parAB* were the PAS and PPS, respectively, most frequently detected. These findings suggest that these systems deserve more attention and further research in order to clearly define their role in the successful dissemination of such plasmids.

Moreover, on integrating all the traits reported for the carbapenemase-producing plasmids found in the present work, most of the strains were shown to respectively harbor different plasmids. Nonetheless, the results obtained from the plasmid characterization suggest that the KPC-2-producing strains *E*. *coli* CAR-1 and *E*. *cloacae* CAR-7 may harbor the same plasmid (i.e, 70-kb IncN plasmid carrying the VagCD system as well as the *bla*_OXA-1_, *aac(6’)-Ib* and *qnrB6* determinants). As these two strains belonged to different species, and taking into consideration the successful plasmid mobilization results for these two strains, it is easy to assume the occurrence of an *in vivo* environmental transference of this plasmid supporting its dissemination ability. This fact would provide means for this resistance determinant to eventually reach a human-adapted bacterial clone endangering the efficacy of the antimicrobial armamentarium.

Overall, the present study reports for the first time the presence of CPE recovered from a river ecosystem in the Barcelona metropolitan area. The results show that several species produce, and sometimes coproduce, different carbapenemases which are carried in different plasmids. No epidemiological relationship between these environmental isolates and the pathogenic strains detected in the clinical setting was found, hence suggesting that they might rather act as reservoirs. Surprisingly, KPC-2 was the most prevalent enzyme in contrast with the clinical situation in our country. These results reinforce the necessity to take into account all the diverse reservoirs encountered, including humans, animals and the environment, in the fight against antimicrobial resistance.

## Supporting information

S1 Table List of primers used in this study. (PDF)
