# Supplementary material for: Carbapenemase-producing enterobacteriaceae recovered from a Spanish river ecosystem
Source: PLoS One. 2017 Apr 5;12(4):e0175246. doi: 10.1371/journal.pone.0175246 (PMC5381907; doi:10.1371/journal.pone.0175246)
Supplement: S1 Table — (PDF) [file pone.0175246.s001.pdf]

Supplementary Table S1. List of primers used in this study.

Genes	Primer name	Primer sequence (5' to 3')	Product size	Temperature	Reference
Resistance					
bla$_{OXA-48}$	OXAC-G48F	TGTRTTAGCCTTATCGGC	678 bp	55 ºC	This study
	OXAC-G48R	TCYAGTTCAACCCAACCG			
bla$_{KPC}$	KPCn-F	CTGTCTTGTCTCTCATGG	784 bp	55 ºC	Modified from (1)
	KPCn-R	TGTCATCCTTGTTAGGCG			
bla$_{IMI}$	IMIn-F	ACAGGCCAATACAAAGGG	606 bp	55 ºC	This study
	IMIn-R	TACGCTAGCACGAATACG			
bla$_{NDM}$	NDM-F	GGCCGTATGAGTGATTGC	775 bp	55 ºC	This study
	NDM-R	TATTATGCACCCGGTCGC			
bla$_{IMP}$	IMP-F	ATAGRGTGGCTTAATTCTC	230 bp	55 ºC	Modified from (2)
	IMP-R	GGTTTAARAAAACAACCACC			
bla$_{VIM}$	VIM-G2F	TGGTCGCATATCGCAACG	500 bp	55 ºC	Modified from (2)
	VIM-G2R	GGCCATTCAGCCAGATCG			
bla$_{TEM}$	TEM-P3	AGTGTCGACTTACCAATGCTTAATCAGT	942bp	55 ºC	(3)
	TEM-P4	AAAGAATTCTAAATACATTCAAATATG			
bla$_{SHV}$	SHV-A	CGCCGGGTTATTCTTATTTGTCGC	1017 bp	60 ºC	(4)
	SHV-B	TCTTTCCGATGCCGCCGCCAGTCA			
bla$_{CTX-M}$	CTX-M-G1F	AAAAATCACTGCGCCAGTTC	415 bp	52 ºC	(5)
	CTX-M-G1R	AGCTTATTCATCGCCACGTT			
	CTX-M-G2F	TTT GCG ATG TGC AGT ACC AGT AA	391 bp	52 ºC	(5)
	CTX-M-G2R	CCA GCG TCA GAT TTT TCA GG			
	CTX-M-G9F	CAA AGA GAG TGC AAC GGA TG	205 bp	52 ºC	(5)

	CTX-M-G9R	ATT GGA AAG CGT TCA TCA CC			
aac(6')-Ib	Aac6-Ib-F	TGCGATGCTCTATGAGTGGCTA	481 bp	55 ºC	This study
	Aac6-Ib-R	CTCGAATGCCTGGCGTGTTT			
qnrA	QnrA-F	CTAATCCGGCAGCACTATTA	661 bp	55 ºC	(6)
	QnrA-R	GGGTATGGATATTATTGATAAAG			
qnrB	QnrB-F	GGCACTGAATTTATCGGC	431 bp	55 ºC	(7)
	QnrB-R	TCCGAATTGGTCAGATCG			
qnrS	QnrS-F	CCTACAATCATACATATCGGC	621bp	55 ºC	(7)
	QnrS-R	GCTTCGAGAATCAGTTCTTGC			
Virulence (Klebsiella)					
fimH	fim-1	GCT CTG GCC GAT AC(C/T) AC(C/G) ACG G	423 bp	55 ºC	(8)
	fim-2	GC(G/A) (A/T)A (G/A)T AAC G(T/C) GCC TGG AAC GG			
mrkD	mrkD-1	TAT (T/C)G(G/T) CTT AAT GGC GCT GG	920 bp	50 ºC	(8)
	mrkD-2	TAA TCG TAC GTC AGG TTA AAG A(C/T)C			
wabG	wabG-F	CGG ACT GGC AGA TCC ATA TC	683 bp	53 ºC	(8)
	wabG-R	ACC ATC GGC CAT TTG ATA GA			
uge	uge-F	GAT CAT CCG GTC TCC CTG TA	534 bp	53 ºC	(9)
	uge-R	TCT TCA CGC CTT CCT TCA CT			
ureA	ureA-F	GCT GAC TTA AGA GAA CGT TAT G	337 bp	55 ºC	(8)
	ureA-R	GAT CAT GGC GCT ACC T(C/T) A			
rmpA	rmpA-F	ACT GGG CTA CCT CTG CTT CA	535 bp	46 ºC	(8)
	rmpA-R	CTT GCA TGA GCC ATC TTT CA			
magA	magA-F	GGT GCT CTT TAC ATC ATT GC	1283 bp	50 ºC	(10)
	magA-R	GCA ATG GCC ATT TGC GTT AG			
wzy	wzy-F	GACCCGATATTCATACTTGACAGAG	641 bp	55 ºC	(11)
	wzy-R	CCTGAAGTAAAATCGTAAATAGATGGC			

kfuBC	kfuB-F1179	GAA GTG ACG CTG TTT CTG GC	797 bp	55 ºC	(12)
	kfuC-R649	TTT CGT GTG GCC AGT GAC TC			
clbB	clbB-F	GATTTGGATACTGGCGATAACCG	579 bp	55 ºC	(13)
	clbB-R	CCATTTCCCGTTTGAGCACAC			
clbN	clbN-F	GTTTTTGCTCGCCAGATAGTCATTC	733 bp	55 ºC	(13)
	clbN-R	CAGTTCGGGTATGTGTGGAAGG			
allS	1416R	CCG TTA GGC AAT CCA GAC	1090 bp	49 ºC	(14)
	336F2	TCT GAT TTA (A/T)CC CAC ATT			
Plasmid addiction systems					
pemK	PemK-up	AAC GAG AAT GGC TGG ATG C	232 bp	54 ºC	(15)
	PemK-low	CCA ACG ACA CCG CAA AGC			
ccdAB	CcdA-up	AGG AAG GGA TGG CTG AGG T	230 bp	54 ºC	(15)
	CcdB-low	GGT AAA GTT CAC GGG AGA C			
relE	RelE-up	AAA AAC CCG ATG GCG ACA G	370 bp	57 ºC	(15)
	RelE-low	TGA TAG ACC AGG CGA AAA C			
parDE	ParD-up	ACG GAC CAG CAG CAC CAG	534 bp	58 ºC	(15)
	ParE-low	AGC CCT TGA GCC TGT CGG			
vagCD	VagC-up	GGG ACC TGG ATT TTG ATG G	210 bp	53 ºC	(15)
	VagD-low	GAG CAG ATG TTG GTG TCG			
hok-sok	Hok-up	AGA TAG CCC CGT AGT AAG TT	203 bp	54 ºC	(15)
	Sok-low	GAT TTT CGT GTC AGA TAA GTG			
pndCA	PndC-up	TCA ATC AAC CAG GGC TCT	140 bp	52 ºC	(15)
	PndA-low	CCT CAC CAT CCA GAC AAA A			
srnBC	SrnB-up	ACT GAT TGT AGC CTC TTC TTT	171 bp	54 ºC	(15)
	SrnC-low	CAC CAC TGT ATT TCC CCT GT			
Plasmid partition systems					

ParMRC-F	GGTTTCTTTCGTCAGCAGCTCACA	1081bp	55 ºC	This study
ParMRC-R	CCGAACAGCTTCAAACGCGAGT			
SopAB-F	GGCACAGCCTCGATGTATCAC	1232bp	55 ºC	This study
SopAB-R	GGCTTGTATAACGCAGGCCA			
ParAB-F	GCTCCGACTGAACCGGGAA	1738bp	55 ºC	This study
ParAB-R	GCGAAAGTATCCGACAGCAA			

References:

1. Poirel L, Walsh TR, Cuvillier V, Nordmann P. Multiplex PCR for detection of acquired carbapenemase genes. Diagn Microbiol Infect Dis. 2011 May;70(1):119–23.

2. Balsalobre LC, Dropa M, Lincopan N, Mamizuka EM, Matté GR, Matté MH. Detection of metallo-β-lactamases-encoding genes in environmental isolates of *Aeromonas hydrophila* and *Aeromonas jandaei*. Lett Appl Microbiol. 2009 Jul;49(1):142–5.

3. Sabaté M, Miró E, Navarro F, Vergés C, Aliaga R, Mirelis B, et al. β-lactamases involved in resistance to broad-spectrum cephalosporins in Escherichia coli and Klebsiella spp. clinical isolates collected between 1994 and 1996, in Barcelona (Spain). J Antimicrob Chemother. 2002;49:989–97.

4. Tracz DM. Increase in ampC promoter strength due to mutations and deletion of the attenuator in a clinical isolate of cefoxitin-resistant Escherichia coli as determined by RT-PCR. J Antimicrob Chemother. 2005 Apr 8;55(5):768–72.

5. Woodford N, Fagan EH, Ellington MJ. Multiplex PCR for rapid detection of genes encoding CTX-M extended-spectrum -lactamases. J Antimicrob Chemother. 2005 Nov 24;57(1):154–5.

6. Wang M, Sahm DF, Jacoby GA, Hooper DC. Emerging Plasmid-Mediated Quinolone Resistance Associated with the qnr Gene in Klebsiella pneumoniae Clinical Isolates in the United States. Antimicrob Agents Chemother. 2004 Apr 1;48(4):1295–9.

7. Lavilla S, Gonzalez-Lopez JJ, Sabate M, Garcia-Fernandez A, Larrosa MN, Bartolome RM, et al. Prevalence of qnr genes among extended-spectrum -lactamase-producing enterobacterial isolates in Barcelona, Spain. J Antimicrob Chemother. 2008;61(2):291–5.

8. Brisse S, Fevre C, Passet V, Issenhuth-Jeanjean S, Tournebize R, Diancourt L, et al. Virulent Clones of Klebsiella pneumoniae: Identification and Evolutionary Scenario Based on Genomic and Phenotypic Characterization. Neyrolles O, editor. PLoS ONE. 2009 Mar 25;4(3):e4982.

9. Regue M, Hita B, Pique N, Izquierdo L, Merino S, Fresno S, et al. A Gene, uge, Is Essential for Klebsiella pneumoniae Virulence. Infect Immun. 2004 Jan 1;72(1):54–61.

10. Fang C-T, Chuang Y-P, Shun C-T, Chang S-C, Wang J-T. A Novel Virulence Gene in *Klebsiella pneumoniae* Strains Causing Primary Liver Abscess and Septic Metastatic Complications. J Exp Med. 2004 Mar 1;199(5):697–705.

11. Turton JF, Baklan H, Siu LK, Kaufmann ME, Pitt TL. Evaluation of a multiplex PCR for detection of serotypes K1, K2 and K5 in *Klebsiella* sp. and comparison of isolates within these serotypes. FEMS Microbiol Lett. 2008 Jul;284(2):247–52.

12. Ma L-C, Fang C-T, Lee C-Z, Shun C-T, Wang J-T. Genomic Heterogeneity in Klebsiella pneumoniae Strains Is Associated with Primary Pyogenic Liver Abscess and Metastatic Infection. J Infect Dis. 2005;192:117–28.

13. Johnson JR, Johnston B, Kuskowski MA, Nougayrede J-P, Oswald E. Molecular Epidemiology and Phylogenetic Distribution of the Escherichia coli pks Genomic Island. J Clin Microbiol. 2008 Dec 1;46(12):3906–11.

14. Chou H-C, Lee C-Z, Ma L-C, Fang C-T, Chang S-C, Wang J-T. Isolation of a Chromosomal Region of Klebsiella pneumoniae Associated with Allantoin Metabolism and Liver Infection. Infect Immun. 2004 Jul 1;72(7):3783–92.

15. Mnif B, Vimont S, Boyd A, Bourit E, Picard B, Branger C, et al. Molecular characterization of addiction systems of plasmids encoding extended-spectrum -lactamases in Escherichia coli. J Antimicrob Chemother. 2010 Aug 1;65(8):1599–603.
